# Synthesis and structure of 9-(di­methyl­amino)-1,10-phenanthrolin-1-ium nitrate

**DOI:** 10.1107/S2056989026006882

**Published:** 2026-07-10

**Authors:** Batirbay Torambetov, Mukhammedaliy Allaniyazov, Jamshid Ashurov, Tuncer Hökelek, Alebel N. Belay, Khudayar I. Hasanov, Narmina A. Guliyeva

**Affiliations:** a National University of Uzbekistan named after Mirzo Ulugbek 4 University St Tashkent 100174 Uzbekistan; b Institute of Bioorganic Chemistry, Academy of Sciences of Uzbekistan, M. Ulugbek St. 83, Tashkent 100125, Uzbekistan; c Hacettepe University, Department of Physics, 06800 Beytepe-Ankara, Türkiye; d Department of Chemistry, Bahir Dar University, PO Box 79, Bahir Dar, Ethiopia; e Azerbaijan Medical University, Scientific Research Centre (SRC), A. Kasumzade St. 14, AZ 1022, Baku, Azerbaijan; f Department of Chemical Engineering, Baku Engineering University, Hasan Aliyev Str. 120, AZ0101, Khirdalan, Absheron, Azerbaijan; University of Aberdeen, United Kingdom

**Keywords:** 1,10-phenanthroline, crystal structure, noncovalent inter­actions

## Abstract

In the title salt, C_14_H_14_N_3_^+^·NO_3_^−^, the cation is almost planar (r.m.s. deviation = 0.014 Å), implying significant conjugation of the lone pair of the tertiary amino group N atom with the aromatic ring system. In the extended structure, N—H⋯O and C—H⋯O hydrogen bonds link the components into infinite chains of alternating cations and anions propagating along the *b*-axis direction. Aromatic π–π stacking inter­actions with centroid–centroid distances of 3.5773 (12) and 3.5889 (12) Å may help to consolidate the packing. Hirshfeld surface analysis revealed that the most important contributions for the crystal packing are from H⋯H (39.2%), H⋯O/O⋯H (31.6%), H⋯C/C⋯H (10.3%) and C⋯C (9.1%) inter­actions.

## Abstract 2

In the title salt, the cation is almost planar, implying significant conjugation of the lone pair of the secondary amino group N atom with the aromatic ring system. In the extended structure, N—H⋯O and C—H⋯O hydrogen bonds link the components into infinite chains of alternating cations and anions propagating along the *b*-axis direction.

## 1. Chemical context

1,10-Phenanthroline and its derivatives are widely used in medicinal, catalysis, materials and coordination chemistry (*e.g*., Queffelec *et al.*, 2024 ▸; Krawiec *et al.*, 2005 ▸; Kumar *et al.*, 2022 ▸). Polyaromatic rings in this class of organic compounds possess rigidity and robustness, which may be significant in the design of functional materials such as catalysts, luminescent coordination scaffolds, supra­molecular aggregates, sensors and theranostics. 1,10-Phenanthroline derivatives are popular ligands in coordination chemistry due to their strong affinities for a wide range of metals with various oxidation states (Naithani *et al.*, 2023 ▸). Substitution of the aromatic ring of 1,10-phenanthroline can be used as an important synthetic strategy towards new materials (Figueiredo *et al.*, 2022 ▸; Tsvetkov *et al.*, 2025 ▸).
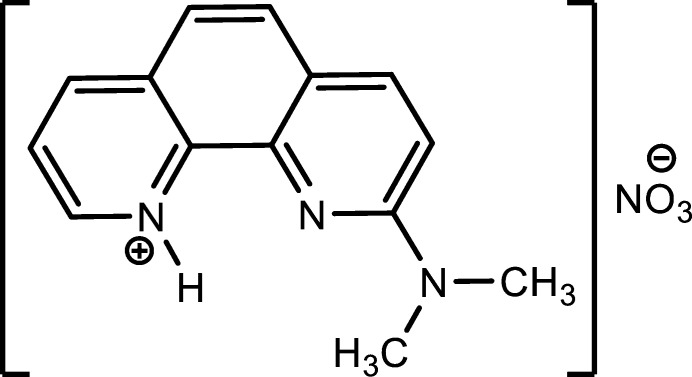


As part of our studies in this area, we now report the synthesis and structure of the title salt, C_14_H_14_N_3_^+^·NO_3_^−^ (**I**).

## 2. Structural commentary

**Figure 1:**
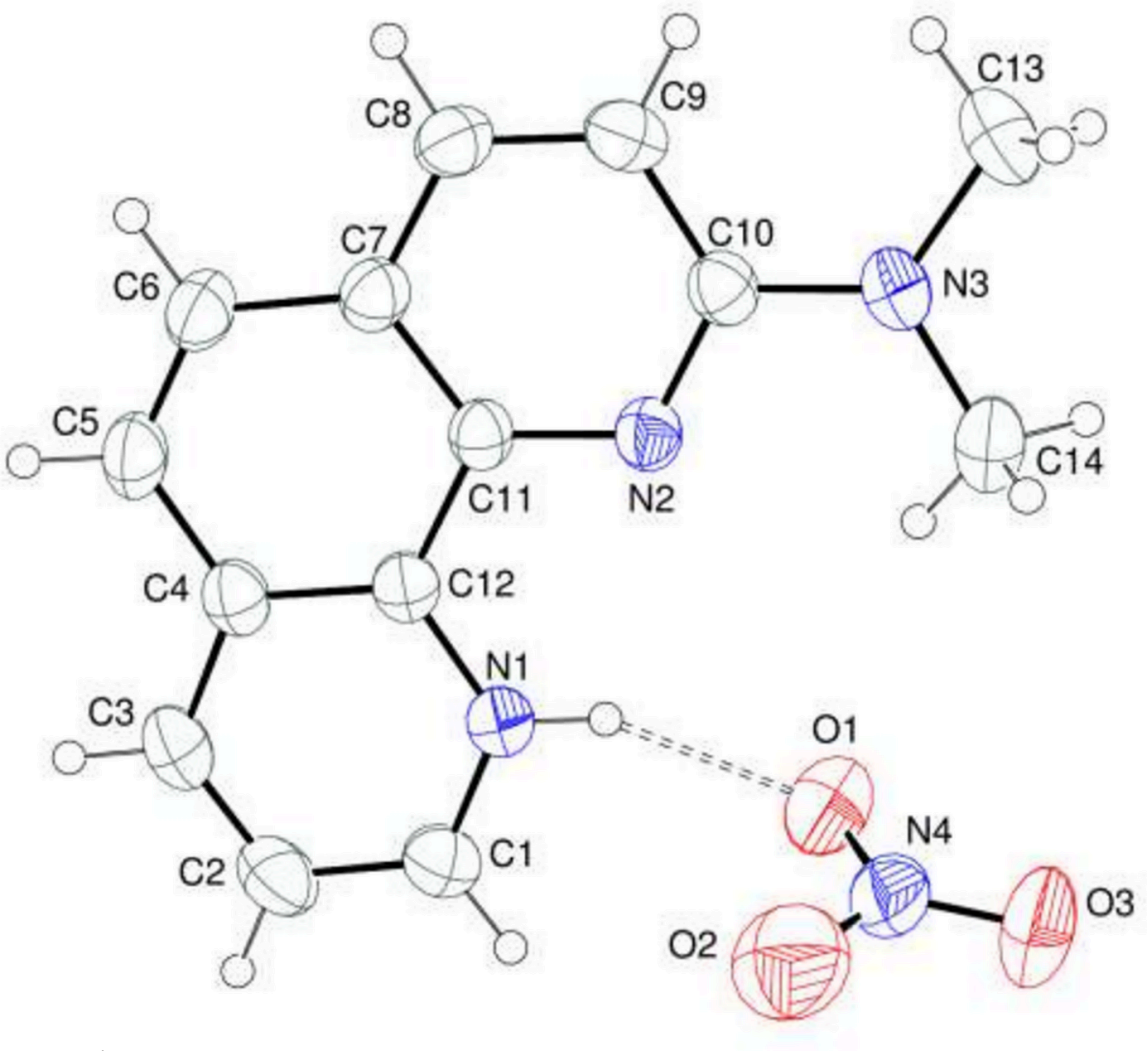
The mol­ecular structure of (**I**) with 50% probability ellipsoids. The N—H⋯O hydrogen bond is shown as a dashed line.

The asymmetric unit of (**I**), which crystallizes in space group *P*2_1_/*n*, consists of one di­methyl­amino­phenanthroline cation and one nitrate counter ion (Fig. 1 ▸). The phenanthroline ring system is almost planar with an r.m.s. deviation of 0.014 Å. Atoms N3, C13 and C14 are displaced by −0.035 (2), −0.001 (3) and −0.011 (3) Å, respectively, away from the best least-squares plane of the phenanthroline ring system. Evidence of their near co-planarity with the ring system is further supported by the C13—N3—C10—N2 [–178.5 (2)°] and C14—N3—C10—N2 [–2.6 (3)°] torsion angles. The C1—N1—C12 [123.41 (18)°] bond angle at the protonated N1 atom of the ring system is significantly enlarged compared to the unprotonated C10—N2—C11 [117.80 (17)°] bond angle, which is consistent with previous studies, e.g., Hensen *et al.* (2000 ▸) and Büyükekşi *et al.* (2019 ▸). The N1—C1 [1.322 (3) Å] and N1—C12 [1.353 (2) Å] bond lengths of the protonated N1 atom are similar to the N2—C10 [1.336 (2) Å] and N2—C11 [1.345 (2) Å] bonds of the non-protonated N2 atom. In the pendant di­methyl­amino group, the N3—C10 [1.357 (3) Å] bond length is significantly shorter than N3—C13 [1.453 (3) Å] and N3—C14 [1.444 (3) Å] bonds, presumably because of resonance-assisted π-conjugation, where the N atom of the di­methyl­amino group acts as a strong electron donor to the aromatic ring system. On the other hand, the C10—N3—C13 [123.3 (2)°] and C14—N3—C13 [115.69 (19)°] bond angles are significantly enlarged and narrowed according to the bond angle of C10—N3—C14 [120.86 (18)°], respectively. In the nitrate counter-ion, the N4—O3 [1.204 (3) Å] bond is significantly shorter than N4—O1 [1.251 (3) Å] and N4—O2 [1.245 (3) Å], possibly due to hydrogen bonding and crystal packing effects, where O1 acts as hydrogen-bond acceptor inter­acting with the protonated N1 atom of the phenanthroline ring system. The O2—N4—O1 [117.5 (2)°] bond angle is significantly narrower than O3—N4—O1 [120.6 (3)°] and O3—N4—O2 [121.8 (3)°].

## 3. Supra­molecular features

**Figure 2:**
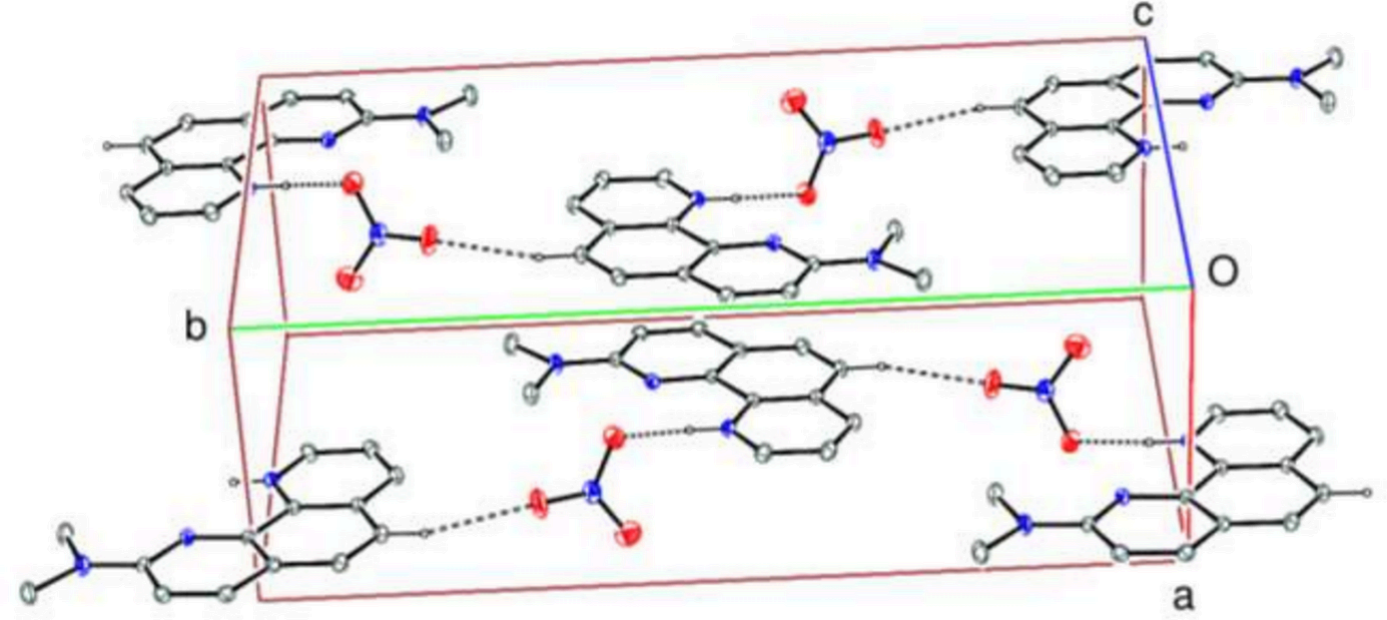
Packing diagram of (**I**) showing N—H⋯O and C—H⋯O hydrogen bonds as dashed lines with the infinite chains propagating along the *b*-axis direction.

In the extended structure, the N1—H1⋯O1 hydrogen bond (Table 1 ▸) links the cation to the anion (Fig. 2 ▸) and a C5—H5⋯O3 hydrogen bond (Table 2 ▸) links the ion pairs into infinite chains propagating along the *b*-axis direction (Fig. 2 ▸). Further, π–π stacking inter­actions between the *A* (N1/C1–C4/C12) and *B* (N2/C7–C11) rings and also between the *B* and *C* (C4–C12) rings of the phenanthroline ring system with centroid–to–centroid distances of 3.5773 (12) Å [α = 1.34 (10)° and slippage = 1.212 Å] and 3.5889 (12) Å [α = 0.84 (9)° and slippage = 1.198 Å], respectively, help to consolidate the packing. No C—H⋯π(ring) inter­actions are observed.

**Figure 3:**
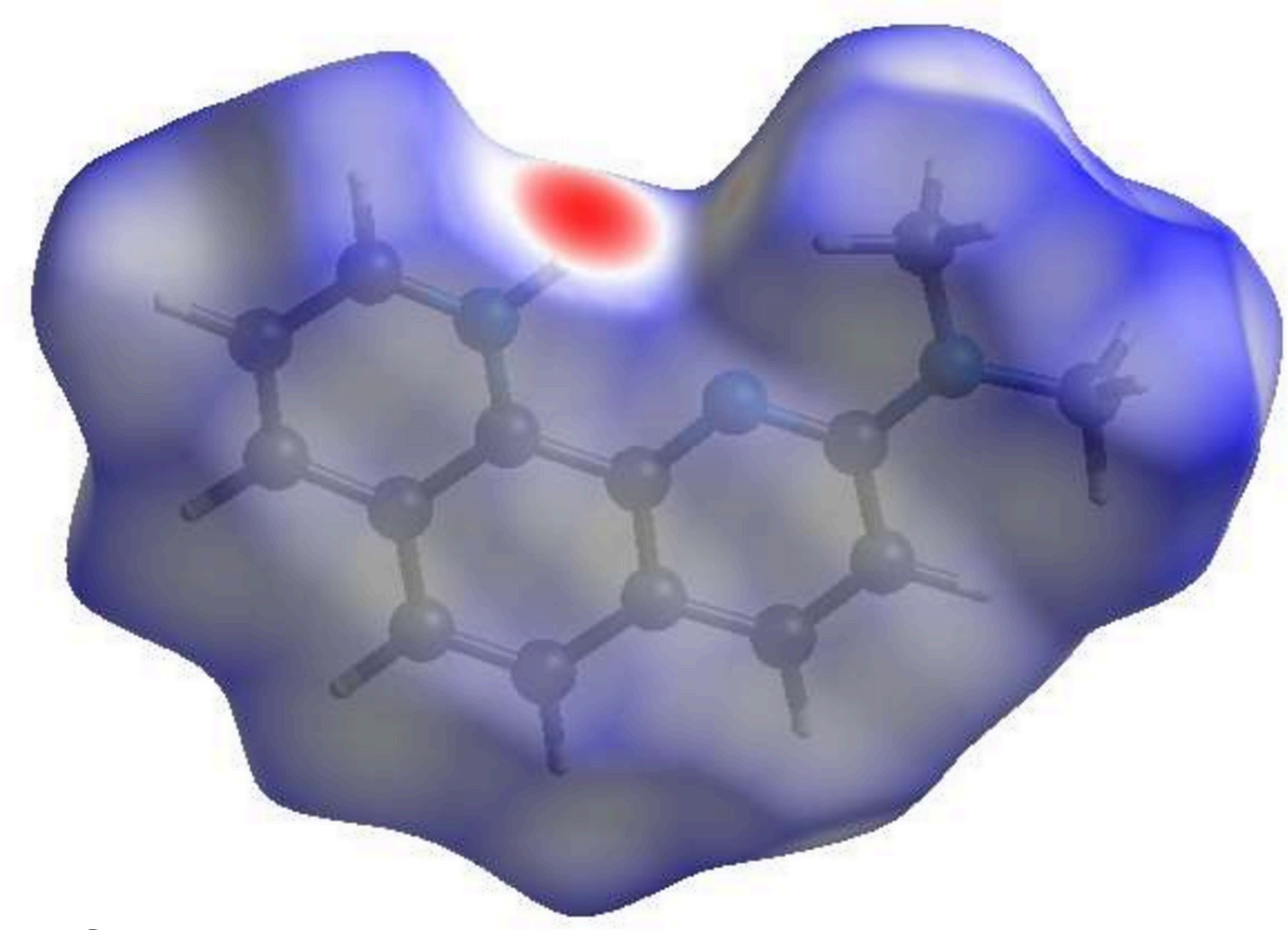
View of the three-dimensional Hirshfeld surface for (**I**) plotted over *d*_norm_ in the range −0.54 to 1.08 a.u.

**Figure 4:**
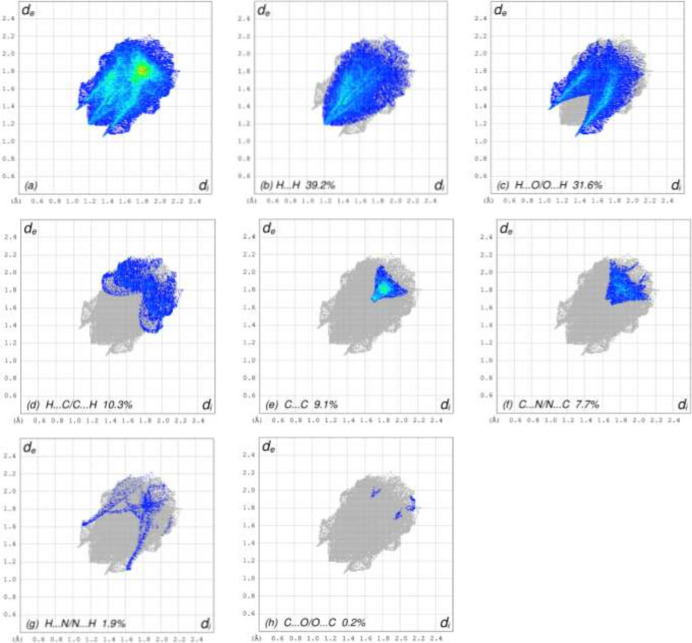
Two-dimensional fingerprint plots for (**I**), showing (*a*) all inter­actions, and delineated (*b*)–(*f*) into different contact types. The *d*_i_ and *d*_e_ values are the closest inter­nal and external distances (in Å) from given points on the Hirshfeld surface.

The inter­molecular inter­actions in the crystal were visualized by carrying out the Hirshfeld surface (HS) analysis using *CrystalExplorer* 17.5 (Spackman *et al.*, 2021 ▸). Fig. 3 ▸ shows the Hirshfeld surface with the red spots corresponding to the hydrogen-bond donors and acceptors noted above. The overall two-dimensional fingerprint plot is shown in Fig. 4 ▸*a* and those delineated into the different contact types are illustrated in Fig. 4 ▸ (*b*)–(*h*), respectively. According to the two-dimensional fingerprint plots the H⋯H, H⋯O/O⋯H, H⋯C/C⋯H and C⋯C contacts make the most significant contributions to the HS, at 39.2%, 31.6%, 10.3% and 9.1%, respectively.

## 4. Database survey

A search of the Cambridge Structural Database (CSD, Version 5.45, updated September 2024; Groom *et al.*, 2016 ▸) identified eight compounds with close structural similarity to (**I**). These include: 1,10-phenanthrolin-1-ium-6-sulfonate hydrogen peroxide, C_12_H_8_N_2_O_3_S·H_2_O_2_ (CSD refcode BIPZUZ; Bezzubov *et al.*, 2023 ▸), 1,10-phenanthrolin-1-ium chloride, C_12_H_9_N_2_^+^·Cl^−^ (CUZDIK; Hensen *et al.*, 2000 ▸), 2,9-dimethyl-1,10-phenanthrolin-1-ium picrate, C_6_H_2_N_3_O_7_^−^·C_14_H_13_N_2_^+^ (CIYPIL; Chan *et al.*, 2014 ▸), 6-ethynyl-1,10-phenanthrolin-1-ium tri­fluoro­methane­sulfonate, CF_3_O_3_S^−^·C_14_H_9_N_2_^+^ (DILPIA; Doistau *et al.*, 2018 ▸), 2-amino-1,10-phenanthrolin-1-ium chloride, C_12_H_10_N_3_^+^·Cl^−^ (LUZZAL; Büyükekşi *et al.*, 2019 ▸), 9-phenyl-1,10-phenanthrolin-1-ium tri­fluoro­methane­sulfonate, C_18_H_13_N_2_^+^·CF_3_SO_3_^−^ (NIYSUL; Krause *et al.*, 2014 ▸), 9-phenyl-1,10-phenanthrolin-1-ium tetra­chloro­gold(III), C_18_H_13_N_2_·AuCl_4_ (NIYTAS; Krause *et al.*, 2014 ▸) and 1,10-phenanthrolin-1-ium tetra­fluoro­borate, C_12_H_9_N_2_^+^·BF_4_^−^ (WUJWUX; Ghorai *et al.*, 2024 ▸).

## 5. Synthesis and crystallization

**Figure 5:**
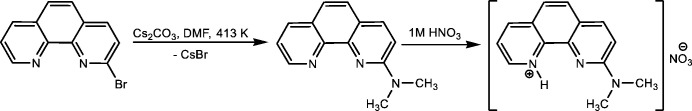
Synthesis of (**I**).

2-Bromo-1,10-phenanthroline (2.59 g, 10 mmol) and Cs_2_CO_3_ (3.25 g, 10 mmol) were dissolved in *N*,*N*-di­methyl­formide (DMF) (75 ml), and the resulting mixture was heated with stirring at 413 K for 6 h (Fig. 5 ▸). After completion of the reaction, the solvent was removed under reduced pressure. The residue was washed with ethyl acetate and water. The neutral mol­ecule was isolated from the ethyl acetate fraction after concentration, giving a yield of 60%. An equimolar amount of 1 *M* aqueous HNO_3_ was added dropwise, with stirring, to an ethano­lic solution and the resulting solution was left to crystallize at room temperature. Colorless crystals of (**I**) were obtained after 6 days. Yield: 65%. Analysis (%) for C_14_H_14_N_4_O_3_, calculated (obtained): C 58.74 (58.70), H 4.93 (4.89), N 19.57 (19.52). ^1^H NMR (400 MHz, DMSO-*d*_6_, ppm): δ 3.19 (6H, NMe_2_), 7.57–9.20 (7H). ^13^C{^1^H} NMR (100 MHz, DMSO-*d*_6_, ppm): δ 42.3, 111.7, 122.7, 123.8, 125.5, 126.9, 128.4, 138.2, 143.5, 144.9, 146.1, 150.4, 157.3.

## 6. Refinement

Crystal data, data collection and structure refinement details are summarized in Table 2 ▸. The N-bound H atom was located from a difference-Fourier map and refined isotropically. The C-bound H-atom positions were calculated geometrically at distances of 0.93–0.96 Å and refined using a riding model by applying the constraint *U*_iso_(H) = *k* × U_eq_ (C), where *k* = 1.2 for aromatic CH hydrogen atoms and *k* = 1.5 for methyl hydrogen atoms.

## Supplementary Material

Crystal structure: contains datablock(s) I, global. DOI: 10.1107/S2056989026006882/hb8230sup1.cif

Structure factors: contains datablock(s) I. DOI: 10.1107/S2056989026006882/hb8230Isup2.hkl

CCDC reference: 2566614

Additional supporting information:  crystallographic information; 3D view; checkCIF report

## Figures and Tables

**Table 1:** Hydrogen-bond geometry (Å, °)

*D*—H⋯*A*	*D*—H	H⋯*A*	*D*⋯*A*	*D*—H⋯*A*
N1—H1⋯O1	0.86 (1)	2.04 (2)	2.819 (3)	150 (3)
C5—H5⋯O3^i^	0.93	2.55	3.407 (3)	154

Symmetry code: (i) 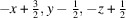.

**Table 2:** Experimental details

Crystal data	
Chemical formula	C_14_H_14_N_3_^+^·NO_3_^−^
*M*_r_	286.29
Crystal system, space group	Monoclinic, *P*2_1_/*n*
Temperature (K)	293
*a*, *b*, *c* (Å)	6.7438 (3), 19.8224 (7), 9.8185 (4)
β (°)	94.762 (3)
*V* (Å^3^)	1307.99 (9)
*Z*	4
Radiation type	Cu *K*α
μ (mm^−1^)	0.88
Crystal size (mm)	0.2 × 0.16 × 0.14

Data collection	
Diffractometer	XtaLAB Synergy, Single source at home/near, HyPix-Bantam
Absorption correction	Multi-scan (*CrysAlis PRO*; Rigaku OD, 2026 ▸)
*T*_min_, *T*_max_	0.243, 1.000
No. of measured, independent and observed [*I* > 2σ(*I*)] reflections	12223, 2527, 1881
*R*_int_	0.039
(sin θ/λ)_max_ (Å^−1^)	0.615

Refinement	
*R*[*F*^2^ > 2σ(*F*^2^)], *wR*(*F*^2^), *S*	0.059, 0.184, 1.07
No. of reflections	2527
No. of parameters	196
No. of restraints	1
H-atom treatment	H atoms treated by a mixture of independent and constrained refinement
Δρ_max_, Δρ_min_ (e Å^−3^)	0.25, −0.20

Computer programs: *CrysAlis PRO* (Rigaku OD, 2026 ▸), *SHELXT* (Sheldrick, 2015*a* ▸), *SHELXL2016/6* (Sheldrick, 2015*b* ▸) and *OLEX2* (Dolomanov *et al.*, 2009 ▸).

## supplementary crystallographic information

### 9-(Dimethylamino)-1,10-phenanthrolin-1-ium nitrate Crystal data

**Table:**

C_14_H_14_N_3_^+^·NO_3_^−^	*F*(000) = 600
*M**_r_* = 286.29	*D*_x_ = 1.454 Mg m^−^^3^
Monoclinic, *P*2_1_/*n*	Cu *K*α radiation, λ = 1.54184 Å
*a* = 6.7438 (3) Å	Cell parameters from 4514 reflections
*b* = 19.8224 (7) Å	θ = 4.5–71.4°
*c* = 9.8185 (4) Å	µ = 0.88 mm^−^^1^
β = 94.762 (3)°	*T* = 293 K
*V* = 1307.99 (9) Å^3^	Block, colorless
*Z* = 4	0.2 × 0.16 × 0.14 mm

### 9-(Dimethylamino)-1,10-phenanthrolin-1-ium nitrate Data collection

**Table:**

XtaLAB Synergy, Single source at home/near, HyPix-Bantam diffractometer	2527 independent reflections
Radiation source: micro-focus sealed X-ray tube, PhotonJet (Cu) X-ray Source	1881 reflections with *I* > 2σ(*I*)
Mirror monochromator	*R*_int_ = 0.039
Detector resolution: 10.0000 pixels mm^-1^	θ_max_ = 71.6°, θ_min_ = 4.5°
ω scans	*h* = −8→8
Absorption correction: multi-scan (CrysAlisPro; Rigaku OD, 2026)	*k* = −22→24
*T*_min_ = 0.243, *T*_max_ = 1.000	*l* = −12→12
12223 measured reflections	

### 9-(Dimethylamino)-1,10-phenanthrolin-1-ium nitrate Refinement

**Table:**

Refinement on *F*^2^	1 restraint
Least-squares matrix: full	Hydrogen site location: mixed
*R*[*F*^2^ > 2σ(*F*^2^)] = 0.059	H atoms treated by a mixture of independent and constrained refinement
*wR*(*F*^2^) = 0.184	*w* = 1/[σ^2^(*F*_o_^2^) + (0.1045*P*)^2^ + 0.2504*P*] where *P* = (*F*_o_^2^ + 2*F*_c_^2^)/3
*S* = 1.07	(Δ/σ)_max_ = 0.001
2527 reflections	Δρ_max_ = 0.25 e Å^−^^3^
196 parameters	Δρ_min_ = −0.20 e Å^−^^3^

### 9-(Dimethylamino)-1,10-phenanthrolin-1-ium nitrate Special details

**Table:**

Geometry. All esds (except the esd in the dihedral angle between two l.s. planes) are estimated using the full covariance matrix. The cell esds are taken into account individually in the estimation of esds in distances, angles and torsion angles; correlations between esds in cell parameters are only used when they are defined by crystal symmetry. An approximate (isotropic) treatment of cell esds is used for estimating esds involving l.s. planes.

### 9-(Dimethylamino)-1,10-phenanthrolin-1-ium nitrate Fractional atomic coordinates and isotropic or equivalent isotropic displacement parameters (Å^2^)

**Table:**

	*x*	*y*	*z*	*U*_iso_*/*U*_eq_	
O1	0.5907 (4)	0.61057 (11)	0.1377 (2)	0.0922 (7)	
O2	0.8825 (4)	0.60068 (15)	0.0709 (3)	0.1135 (8)	
O3	0.7228 (5)	0.69350 (10)	0.0406 (3)	0.1222 (10)	
N4	0.7340 (4)	0.63673 (11)	0.0841 (2)	0.0707 (6)	
N1	0.7044 (3)	0.48753 (9)	0.26544 (17)	0.0469 (4)	
N2	0.7379 (2)	0.57058 (8)	0.48756 (17)	0.0441 (4)	
N3	0.7401 (3)	0.67846 (9)	0.5727 (2)	0.0595 (5)	
C1	0.6868 (4)	0.45120 (12)	0.1522 (2)	0.0575 (6)	
H1A	0.668242	0.472441	0.067649	0.069*	
C2	0.6959 (4)	0.38109 (12)	0.1595 (3)	0.0637 (6)	
H2	0.683511	0.355146	0.080373	0.076*	
C3	0.7233 (3)	0.35116 (11)	0.2844 (3)	0.0574 (6)	
H3	0.729509	0.304362	0.289935	0.069*	
C4	0.7424 (3)	0.38943 (10)	0.4051 (2)	0.0469 (5)	
C5	0.7720 (3)	0.36087 (11)	0.5380 (3)	0.0556 (6)	
H5	0.778823	0.314270	0.548464	0.067*	
C6	0.7903 (3)	0.40108 (11)	0.6490 (2)	0.0553 (6)	
H6	0.809475	0.381530	0.735149	0.066*	
C7	0.7810 (3)	0.47261 (10)	0.6381 (2)	0.0459 (5)	
C8	0.8034 (3)	0.51706 (11)	0.7505 (2)	0.0509 (5)	
H8	0.826659	0.499836	0.838442	0.061*	
C9	0.7912 (3)	0.58476 (11)	0.7312 (2)	0.0518 (5)	
H9	0.805535	0.613818	0.805706	0.062*	
C10	0.7564 (3)	0.61119 (10)	0.5964 (2)	0.0444 (5)	
C11	0.7504 (3)	0.50365 (9)	0.50939 (19)	0.0395 (4)	
C12	0.7314 (3)	0.46029 (9)	0.39193 (19)	0.0423 (5)	
C13	0.7615 (5)	0.72889 (12)	0.6803 (3)	0.0765 (8)	
H13A	0.758735	0.707236	0.767585	0.115*	
H13B	0.654123	0.760685	0.668229	0.115*	
H13C	0.885878	0.752077	0.676224	0.115*	
C14	0.7127 (4)	0.70437 (12)	0.4350 (3)	0.0714 (7)	
H14A	0.838078	0.719632	0.406935	0.107*	
H14B	0.620567	0.741374	0.431959	0.107*	
H14C	0.661062	0.669325	0.374545	0.107*	
H1	0.693 (4)	0.5304 (6)	0.254 (3)	0.074 (8)*	

### 9-(Dimethylamino)-1,10-phenanthrolin-1-ium nitrate Atomic displacement parameters (Å^2^)

**Table:**

	*U*^11^	*U*^22^	*U*^33^	*U*^12^	*U*^13^	*U*^23^
O1	0.1127 (17)	0.0782 (13)	0.0871 (14)	0.0041 (12)	0.0165 (13)	0.0218 (11)
O2	0.1078 (18)	0.129 (2)	0.1037 (18)	0.0283 (16)	0.0054 (14)	0.0172 (15)
O3	0.195 (3)	0.0546 (12)	0.1178 (19)	−0.0092 (14)	0.0194 (18)	0.0274 (12)
N4	0.1038 (18)	0.0568 (12)	0.0496 (11)	0.0007 (12)	−0.0039 (11)	0.0033 (9)
N1	0.0551 (10)	0.0411 (9)	0.0444 (9)	0.0024 (8)	0.0039 (7)	−0.0003 (7)
N2	0.0478 (9)	0.0373 (9)	0.0474 (9)	−0.0018 (7)	0.0061 (7)	0.0013 (7)
N3	0.0833 (14)	0.0369 (9)	0.0583 (11)	−0.0038 (9)	0.0051 (9)	−0.0057 (8)
C1	0.0674 (14)	0.0557 (13)	0.0488 (12)	0.0032 (11)	0.0023 (10)	−0.0064 (9)
C2	0.0729 (16)	0.0556 (13)	0.0621 (14)	0.0023 (11)	0.0029 (11)	−0.0181 (11)
C3	0.0615 (14)	0.0402 (11)	0.0711 (15)	0.0010 (9)	0.0082 (11)	−0.0092 (10)
C4	0.0445 (11)	0.0392 (10)	0.0581 (12)	−0.0008 (8)	0.0101 (9)	−0.0009 (9)
C5	0.0624 (13)	0.0371 (10)	0.0684 (14)	0.0015 (9)	0.0120 (11)	0.0092 (10)
C6	0.0651 (14)	0.0461 (11)	0.0553 (12)	0.0026 (10)	0.0094 (10)	0.0135 (10)
C7	0.0466 (11)	0.0448 (11)	0.0472 (11)	0.0005 (8)	0.0099 (8)	0.0060 (8)
C8	0.0570 (12)	0.0553 (12)	0.0408 (10)	0.0021 (10)	0.0074 (8)	0.0055 (9)
C9	0.0561 (13)	0.0545 (12)	0.0454 (11)	−0.0035 (10)	0.0082 (9)	−0.0097 (9)
C10	0.0439 (10)	0.0402 (10)	0.0495 (11)	−0.0039 (8)	0.0068 (8)	−0.0029 (8)
C11	0.0378 (10)	0.0360 (9)	0.0451 (10)	−0.0011 (7)	0.0063 (7)	0.0020 (8)
C12	0.0399 (10)	0.0399 (11)	0.0477 (11)	0.0000 (8)	0.0073 (8)	0.0007 (8)
C13	0.102 (2)	0.0454 (13)	0.0827 (18)	−0.0057 (13)	0.0089 (15)	−0.0187 (12)
C14	0.0960 (19)	0.0424 (12)	0.0753 (16)	0.0012 (12)	0.0042 (14)	0.0082 (11)

### 9-(Dimethylamino)-1,10-phenanthrolin-1-ium nitrate Geometric parameters (Å, º)

**Table:**

O1—N4	1.251 (3)	C4—C12	1.412 (3)
O2—N4	1.245 (3)	C5—H5	0.9300
O3—N4	1.204 (3)	C5—C6	1.347 (3)
N1—C1	1.322 (3)	C6—H6	0.9300
N1—C12	1.353 (2)	C6—C7	1.423 (3)
N1—H1	0.861 (10)	C7—C8	1.410 (3)
N2—C10	1.336 (2)	C7—C11	1.405 (3)
N2—C11	1.345 (2)	C8—H8	0.9300
N3—C10	1.357 (3)	C8—C9	1.357 (3)
N3—C13	1.453 (3)	C9—H9	0.9300
N3—C14	1.444 (3)	C9—C10	1.424 (3)
C1—H1A	0.9300	C11—C12	1.435 (3)
C1—C2	1.393 (3)	C13—H13A	0.9600
C2—H2	0.9300	C13—H13B	0.9600
C2—C3	1.361 (4)	C13—H13C	0.9600
C3—H3	0.9300	C14—H14A	0.9600
C3—C4	1.404 (3)	C14—H14B	0.9600
C4—C5	1.421 (3)	C14—H14C	0.9600
			
O2—N4—O1	117.5 (2)	C11—C7—C6	120.40 (19)
O3—N4—O1	120.6 (3)	C11—C7—C8	115.35 (18)
O3—N4—O2	121.8 (3)	C7—C8—H8	119.8
C1—N1—C12	123.41 (18)	C9—C8—C7	120.49 (19)
C1—N1—H1	115.1 (19)	C9—C8—H8	119.8
C12—N1—H1	121.4 (19)	C8—C9—H9	120.1
C10—N2—C11	117.80 (17)	C8—C9—C10	119.80 (19)
C10—N3—C13	123.3 (2)	C10—C9—H9	120.1
C10—N3—C14	120.86 (18)	N2—C10—N3	117.00 (18)
C14—N3—C13	115.69 (19)	N2—C10—C9	121.26 (18)
N1—C1—H1A	120.0	N3—C10—C9	121.74 (18)
N1—C1—C2	120.0 (2)	N2—C11—C7	125.28 (17)
C2—C1—H1A	120.0	N2—C11—C12	117.53 (16)
C1—C2—H2	120.5	C7—C11—C12	117.19 (17)
C3—C2—C1	118.9 (2)	N1—C12—C4	118.91 (17)
C3—C2—H2	120.5	N1—C12—C11	119.66 (17)
C2—C3—H3	119.3	C4—C12—C11	121.42 (17)
C2—C3—C4	121.4 (2)	N3—C13—H13A	109.5
C4—C3—H3	119.3	N3—C13—H13B	109.5
C3—C4—C5	123.77 (19)	N3—C13—H13C	109.5
C3—C4—C12	117.35 (19)	H13A—C13—H13B	109.5
C12—C4—C5	118.88 (19)	H13A—C13—H13C	109.5
C4—C5—H5	119.9	H13B—C13—H13C	109.5
C6—C5—C4	120.20 (19)	N3—C14—H14A	109.5
C6—C5—H5	119.9	N3—C14—H14B	109.5
C5—C6—H6	119.0	N3—C14—H14C	109.5
C5—C6—C7	121.9 (2)	H14A—C14—H14B	109.5
C7—C6—H6	119.0	H14A—C14—H14C	109.5
C8—C7—C6	124.24 (19)	H14B—C14—H14C	109.5
			
N1—C1—C2—C3	−0.1 (4)	C7—C8—C9—C10	−0.3 (3)
N2—C11—C12—N1	0.7 (3)	C7—C11—C12—N1	−179.14 (17)
N2—C11—C12—C4	−179.94 (17)	C7—C11—C12—C4	0.2 (3)
C1—N1—C12—C4	0.2 (3)	C8—C7—C11—N2	−1.2 (3)
C1—N1—C12—C11	179.61 (19)	C8—C7—C11—C12	178.60 (17)
C1—C2—C3—C4	0.0 (4)	C8—C9—C10—N2	−0.9 (3)
C2—C3—C4—C5	−179.7 (2)	C8—C9—C10—N3	179.0 (2)
C2—C3—C4—C12	0.2 (3)	C10—N2—C11—C7	0.1 (3)
C3—C4—C5—C6	179.6 (2)	C10—N2—C11—C12	−179.69 (16)
C3—C4—C12—N1	−0.3 (3)	C11—N2—C10—N3	−178.92 (18)
C3—C4—C12—C11	−179.66 (18)	C11—N2—C10—C9	1.0 (3)
C4—C5—C6—C7	0.0 (3)	C11—C7—C8—C9	1.2 (3)
C5—C4—C12—N1	179.62 (18)	C12—N1—C1—C2	−0.1 (3)
C5—C4—C12—C11	0.3 (3)	C12—C4—C5—C6	−0.4 (3)
C5—C6—C7—C8	−178.6 (2)	C13—N3—C10—N2	−178.5 (2)
C5—C6—C7—C11	0.5 (3)	C13—N3—C10—C9	1.6 (3)
C6—C7—C8—C9	−179.6 (2)	C14—N3—C10—N2	−2.6 (3)
C6—C7—C11—N2	179.55 (18)	C14—N3—C10—C9	177.5 (2)
C6—C7—C11—C12	−0.6 (3)		

### 9-(Dimethylamino)-1,10-phenanthrolin-1-ium nitrate Hydrogen-bond geometry (Å, º)

**Table:**

*D*—H···*A*	*D*—H	H···*A*	*D*···*A*	*D*—H···*A*
N1—H1···O1	0.86 (1)	2.04 (2)	2.819 (3)	150 (3)
C5—H5···O3^i^	0.93	2.55	3.407 (3)	154

Symmetry code: (i) −*x*+3/2, *y*−1/2, −*z*+1/2.
